# Knowledge and attitudes towards use of long acting reversible contraceptives among women of reproductive age in Lubaga division, Kampala district, Uganda

**DOI:** 10.1186/1756-0500-7-153

**Published:** 2014-03-17

**Authors:** Ronald Anguzu, Raymond Tweheyo, Juliet N Sekandi, Vivian Zalwango, Christine Muhumuza, Suzan Tusiime, David Serwadda

**Affiliations:** 1 School of Public Health, Makerere University, Kampala, Uganda; 2 College of Public Health, University of Georgia, Athens, Georgia, USA; 3 Faculty of Health and Medical Sciences, University of Manchester, Manchester, UK

**Keywords:** Family planning, Long-acting reversible contraceptive (LARC), Injectable, Implant, IUD, Knowledge, Attitudes

## Abstract

**Background:**

Uganda has one of the highest total fertility rates globally and in Sub-Saharan Africa. Her high fertility is mainly attributed to the high unmet need for family planning. Use of Long-acting reversible contraceptives (LARC) is low (13%) in Uganda yet they are the most cost-effective contraceptives. This study aimed to assess the reproductive aged women’s knowledge, attitudes, and factors associated with use of LARC.

**Methods:**

A cross-sectional study was conducted involving 565 women (15–49 years) attending private and public health facilities in Lubaga division, Kampala district. Semi-structured questionnaires were used to measure knowledge, attitudes and factors associated with use of LARC; Intra-Uterine Devices, Implants and Injectables. The outcome variable was current use of LARC. A generalized linear regression model was run in STATA version12.0. Prevalence Risk Ratios for associations between current LARC use and independent factors were obtained and regarded significant at 95% CI with *p* < 0.05.

**Results:**

Mean age (SD) and current use of LARC was 26.34 (5.35) and 31.7% respectively. Factors associated with current use of LARC were; previous use adj.PRR 2.89; (95% CI 2.29, 3.81), knowledge of implant administration site adj.PRR 1.83; (95% CI 1.17, 2.87), and perception that; male partner decisions positively influence their contraceptive choices adj.PRR 1.49; (95% CI 1.18, 1.88). Contrary, perception that LARC should be used by married women was negatively associated with use of LARC adj.PRR 0.63; (95% CI 0.44, 0.90).

**Conclusion:**

Knowledge about site of administration, previous use of LARC and women’s attitude that male partners’ choice influence their contraceptive decisions were positively associated with current use of LARC. Contrary, the attitude that LARC was for married women was negatively associated with its use. This study suggests a need to strengthen client education about LARC to dispel possible myths and to consider integrating male partner’s decision making in contraceptive choices for women.

## Background

Uganda’s total fertility rate (TFR) at 6.2 is one of the highest in sub-Saharan Africa and globally [1]. Uganda more so, has a high maternal mortality ratio (MMR) at 435 maternal deaths per 100,000 live births and an infant mortality rate (IMR) of 54 per 1,000 live births per year [2]. High maternal morbidity and mortality could partly be attributed to unintended pregnancies, short birth intervals and higher risk of obstetric and newborn complications associated with low contraceptive use [3]. About 44% of pregnancies in Uganda are unintended [2] with occurrence of unsafe abortions estimated at 62 per 1,000 women aged 15–49 years [4]. These undesirable maternal and child health outcomes associated with high TFR could be substantially reduced by meeting the family planning (FP) needs of women in developing countries [5]. Provision of highly efficacious family planning (FP) services contributes to a reduction in maternal mortality by lowering the risk of maternal death per birth hence preventing high-risk and high-parity births [6]. It also offers individuals and couples’ ability to anticipate and attain the desired number of children by birth spacing and timing [7].

Use of long acting reversible methods is proposed as a strategy to reverse undesirable maternal health consequences in developing countries [8,9]. Scientific evidence has determined implant and copper-bearing Intra-Uterine Device (IUD) contraceptives to be highly effective and well tolerated [10,11]. Similarly, injectables and implants are proven to be safe, effective and reversible contraceptive options [12,13]. Despite evidence of LARC effectiveness and safety, actual uptake in resource-poor settings like Uganda is low, and is possibly affected by several factors such as knowledge and general awareness of contraceptive methods [14], access to different contraceptive methods, user characteristics, technology [15] and socio-economic status [16].

Currently in Uganda, the contraceptive prevalence rate (CPR) is 30% and LARC prevalence is as low as 13% among all women [2,17]. The prevalence of IUD use is lowest at 0.4%, implants as low as 1.9% and injectable contraceptives at 10.7% [2]. Kampala district, the study site, has a LARC prevalence of 22.7% with implant (1.6%), IUD (1.8%) and injectables (19.3%) [2]. This study set out to determine the reasons for low use of LARC in Lubaga division, Kampala, particularly assess knowledge and attitudes among women of reproductive age use of LARC attending reproductive health services.

## Methods

A health facility-based, cross-sectional study was conducted for a period of nine weeks between March and April 2012 in Lubaga division. Lubaga is one of five administrative divisions in Kampala district, the capital of Uganda. The total population of the area is 411,900 with 53.6% females [18] and about 92% lives in peri-urban locations [19]. Lubaga division’s health structure is currently comprised of 124 registered health facilities and these are in three categories; 2 Public facilities, 120 private for profit and 2 Private Not-For-Profit (PNFP) which all provided reproductive health services including LARC services. The participants were both FP users and non-users who were females 15–49 years attending any reproductive health service (i.e. family planning, post-natal and outpatient clinics) in Lubaga division. A family planning user was defined as a woman who at the time of the interview was currently using any contraceptive method [2]. A non-FP user was defined as a woman who at the time of the interview had never used any contraceptive or had previously used a contraceptive but discontinued its use.

Data was entered, cleaned using Epi info 3.5.1 and statistical analysis done with STATA Version12.0. Data was collected using semi-structured interviewer administered questionnaires which contained information on independent variables. During administration of interviews, the respondents were not prompted with any contraceptive pictorials or FP product names. The outcome variable was binary i.e. current user or non-user of LARC methods. Prior to data collection, questionnaires were pre-tested at a non-participating health centre III in Nakawa division in Kampala that was purposively selected due to similarity of participants’ socio-cultural characteristics in order to determine their suitability for collecting study data and then editing of the data collection tools appropriately. Field editing of questionnaires was conducted by the supervisors on daily basis to check for completeness.

### Sample size

The modified Kish, Leslie formula (1965) was used to estimate the sample size [20]. A total of 565 was computed using a 95% confidence interval, 3.5% precision, 20.5% prevalence for current use of LARC in Kampala district [21] and 9% of the sample size used to compensate for non-response [22]. Using probability proportionate-to-size of daily FP clinic attendance by category of health facility, 20 health facilities were selected. Among the total of 20 health facilities selected, 16 private clinics were randomly selected and 2 PNFP’s and 2 Public health facilities were purposively selected respectively. Probability proportionate-to-size sampling was used to select the number of participants to be recruited from each health facility. At each health facility, the first respondent from each clinic (FP, PNC and outpatient clinics) was selected randomly. The subsequent respondents were selected systematically from the daily clinic attendance list until the required respondents were selected.

### Study measurements

The data was collected using a semi-structured questionnaire developed, piloted and translated into the predominant local language (Luganda) by the research team. Information on participants’ socio-demographic characteristics, LARC knowledge attitudes towards use of LARC was collected.

### Knowledge of LARC

Participants’ knowledge was measured by the number of correct responses to 6 unprompted questions. The first three unprompted questions asked were on knowledge about duration of effective protection from pregnancy of IUD, implant and injectable contraceptives. The next three unprompted questions were on knowledge of the site of administration of IUD, implant and injectable contraceptives. A binary response to each question of yes or no was elicited.

### Attitudes towards LARC

The items on attitude of participants towards use of LARC were scored using a 5-point likert scale with 5 responses. The responses were categorized as; ‘strongly agree’, ‘agree’, ‘not sure’, disagree’ and ‘strongly disagree’. These 5 responses where then collapsed into binary variables; agree and disagree. ‘Agree’ for those who responded ‘agree’ or ‘strongly agree’ and ‘disagree’ for those who responded ‘disagree’, ‘strongly disagree’ or ‘not sure’. This measurement is similar to a study in North Ethiopia which measured knowledge and attitudes towards long-acting and Permanent (LAPM) contraceptives [23].

### Statistical analysis

Double data entry and cleaning was done in Epi info 3.5.1 which was then exported to STATA version 12.0 in order to conduct statistical analyses. Descriptive analysis for summary statistics was conducted for the independent variables. The outcome variable was current use of LARC methods which was a binary categorical variable (yes, no). The study computed a prevalence 31.7% for the outcome variable, which is considered to be high therefore Prevalence Risk Ratios (PRR) were used as the appropriate measure of association [24,25]. The PRR was computed for associations between current use of LARC and independent variables using Generalized Linear Model analysis with robust standard errors. Unadjusted PRR were computed with their corresponding 95% confidence intervals (95% CI). The independent variables with a *p* ≤ 0.10 [26] and biologically plausible factors were included in the multivariable model for multivariable analysis in order to identify their independent predictors of use of LARC. Independent variables identified as statistically significant in the multivariable model were reported as being independently associated with current use of LARC.

### Ethics

Ethical approval to conduct this study was obtained from the Makerere University School of Public Health (MakSPH) Higher Degree Research and Ethics Committee on the 22nd February 2012. Informed consent was obtained from all respondents above 18 years in both verbal and written forms. For study participants who were between 15 to 18 years of age, consent and assent was obtained from their parents/guardians and the respondents respectively. Privacy and confidentiality of respondent information was upheld by the research team. Respondent anonymity was observed through use of questionnaire identification numbers. Permission to carry out this study in Lubaga division was obtained from Lubaga division urban council.

## Results

The Table 1 shows the baseline characteristics of women aged 15 to 49 years in the study. A total of 565 participants were interviewed from twenty health facilities in Lubaga division, Kampala district. The mean age of the respondents was 26.3 years (SD: 5.34). All 565 respondents that were sampled were eligible for the interview. Among the respondents, 72.6% were married, 46.5% had attended secondary education and 39.3% were Catholics. More than half (56.6%) had delivered between 1 to 3 children in their lifetime.

**Table 1:** Characteristics of reproductive aged (15 – 49 years) female respondents in Lubaga division, Mar-Apr 2012

**Variable**	**Frequency, N=565**	**Percentage (%)**
Age*		
15 – 19	34	6.0
20 – 24	194	34.3
25 – 29	211	37.3
30 – 34	78	13.8
35 - 49	48	8.5
**Religion**		
Catholic	222	39.3
Islam	146	25.8
Protestant	152	26.9
Others	45	8.0
**Marital status**		
Single	119	20.0
Married	410	72.6
Divorced/Widowed	42	7.4
**Level of education**		
Primary/Never attended	119	21.1
Secondary	263	46.5
Tertiary	183	32.4
**Number of children delivered**		
0	139	24.6
1 – 3	320	56.6
4+	106	18.8
**Ever had an abortion**		
No	445	78.8
Yes	120	21.2
**Previous use of LARC**		
No	230	40.7
Yes	325	59.3
**Current use of LARC**		
No	386	68.3
Yes	179	31.7

*Range = 15 – 49, mean age (SD) = 26.34 ( 5.35), median (IQR) = 25 (23 – 29).

The proportion of participants currently using LARC methods was 31.7%. Among 565 study participants, about 21% (120 out of 565) reported having ever had an abortion. About 57% (325 of 565) reported having used at least one LARC method prior to current use of LARC at the time of the interview.

Among all study respondents, knowledge of duration of effectiveness of specific LARC methods was generally higher among those currently using LARC methods (Table 2). Among all the study participants, knowledge of effective duration of effectiveness of IUD, implant and injectable contraceptives was 68.5%, 69.9% and 87.4% respectively.

**Table 2:** Knowledge of LARC among women of reproductive age in Lubaga division, 2012 (N = 565)

**Variable**	**Total population, N=565**	**Current use of LARC, N=179**
	**n (%)**	**n (%)**
**Knowledge of LARC**		
**a) Knowledge of duration of protection from pregnancy**		
IUD	387 (68.5)	136 (75.98)
Implant	395 (69.9)	139 (77.65)
Injectable	494 (87.4)	161 (89.94)
**b) Knowledge of site of administration**		
IUD	429 (75.9)	148 (82.68)
Implant	453 (80.2)	158 (88.27)
Injectable	519 (91.9)	171 (95.53)

Knowledge of administration site for IUD, implant and injectables was 75.9%, 80.2% and 91.9% respectively. Knowledge levels of administration site of specific LARC methods were high among current use of LARC i.e. 82.7%, 88.3% and 95.5% for IUD, implant and injectables respectively (Table 2).

In Table 3, nearly all participants 95% (534 out of 565) agreed that LARC methods can effectively prevent the occurrence of pregnancy. Out of the 565 participants, 513 (90.8%) agreed that health workers should explain the contraceptive side effects to them. About one third of respondents, 32.9% (186 out of 565) agreed that use of LARC can cause permanent infertility.

**Table 3:** Attitudes towards use of LARC among respondents in Lubaga division

**Items on attitude**	**Agree**	**Disagree**
	***n *** **(%)**	***n *** **(%)**
LARC effectively prevents occurrence of pregnancy	534 (94.5)	31 (5.5)
LARC methods can cause permanent infertility	186 (32.9)	379 (67.1)
LARC should be used by married women	108 (19.1)	513 (80.9)
Partner should decide your contraceptive method to use	272 (48.1)	293 (51.9)
Health workers should explain contraceptive side effects	513 (90.8)	52 (9.2)

Approximately half (48.1%), 272 of 565 respondents agreed that the contraceptive to use is their partners’ decision.

Table 4 shows bivariate analysis of factors associated with current use of LARC methods. Current use of LARC was about one and a half (1.47) times higher among respondents who had children than those who had none (PRR 1.47; 95% CI 1.03, 2.11; *p* = 0.036). Also, current use of LARC about three times significantly higher among respondents who previously used LARC than those who didn’t previously use LARC (PRR 3.05; 95% CI 2.33, 4.00; *p* = 0.000). Current use of LARC was significantly increased; approximately 1.46 times higher among respondents with knowledge of duration of protection from pregnancy by IUD and implants (PRR 1.46; 95% CI 1.10, 2.02; *p* = 0.006) and (PRR 1.45; 95% CI 1.11, 2.02; *p* = 0.009) respectively than those without knowledge.

**Table 4:** Bivariate analysis of independent variables associated with current use of LARC

**OUTCOME: Current use of LARC**					
**Independent variable**	**Total**	**Current use of LARC, *n *** **(%)**	**Unadjusted PRR**	**(95% CI)**	***p-value***
**Age (years)**					
< 24	228	65 (36.31)	1		
≥ 24	337	114 (63.69)	1.19	(0.92-1.53)	0.187
**No. of children delivered**					
None	113	26 (14.53)	1		
Has children	426	153 (85.47)	1.47	(1.03-2.11)	0.036*
**Religion**					
Non catholic	343	107 (59.78)	1		
Catholic	222	72 (40.2)	1.03	(0.81-1.33)	0.757
**Marital status**					
Never married	113	27 (15.08)	1		
Ever married	452	152 (84.92)	1.41	(0.99-2.01)	0.058
**Previous use of LARC**					
No	325	55 (30.73)	1		
Yes	230	124 (69.27)	3.05	(2.33-4.00)	0.000***
**Occupation**					
Unemployed	301	105 (58.66)	1		
Employed	264	74 (41.34)	0.80	(0.63-1.03)	0.083
**Level of education**					
Never attended	115	40 (22.35)	1		
Attended	446	139 (77.65)	0.90	(0.67-1.19)	0.452
**Abortion**					
No	441	137 (76.54)	1		
Yes	120	42 (23.46)	0.66	(0.34-1.28)	0.217
**Knowledge of duration of protection from pregnancy**					
**IUD**					
No	178	43 (24.02)	1		
Yes	387	136 (75.98)	1.46	(1.10-2.02)	0.006**
**Implant**					
No	170	40 (22.35)	1		
Yes	395	139 (77.65)	1.45	(1.11-2.02)	0.009**
**Injectable**					
No	71	18 (10.06)	1		
Yes	494	161 (89.94)	1.29	(0.85-1.96)	0.240
**Knowledge of site of administration**					
**IUD**					
No	136	31 (17.32)	1		
Yes	429	148 (82.68)	1.51	(1.08-2.12)	0.016*
**Implant**					
No	112	21 (11.73)	1		
Yes	453	158 (88.27)	1.86	(1.24-2.79)	0.003**
**Injectable**					
No	46	8 (4.47)	1		
Yes	519	171 (95.53)	1.89	(0.99-3.61)	0.051
**Attitudes towards use of LARC**					
**LARC should be used by married women**					
Disagree	457	156 (87.15)	1		
Agree	108	23 (12.85)	0.62	(0.43-0.92)	0.016*
**Partner should decide your contraceptive use**					
Disagree	293	70 (39.11)	1		
Agree	272	109 (60.89)	1.68	(1.31-2.16)	0.000***
**LARC prevents pregnancy occurrence**					
Disagree	31	9 (5.03)	1		
Agree	534	170 (94.97)	1.09	(0.62-1.93)	0.749
**Health workers should explain the contraceptive side effects**					
Disagree	52	11 (6.15)	1		
Agree	513	168 (93.85)	1.79	(1.01-3.17)	0.047*
**LARC can cause permanent infertility**					
Disagree	379	112 (62.57)	1		
Agree	186	67 (37.43)	1.22	(0.95-1.56)	0.116

Statistically significant at *p < 0.05, **p < 0.01 ***p < 0.001.

On the other hand, current use of LARC methods was significantly reduced if the respondents agreed that LARC should be used by married women (PRR 0.62; 95% CI 0.43, 0.92; *p* = 0.016).

After adjusting for confounding at multivariable analysis (Table 5), factors that remained significantly associated with current use of LARC were: previous use of LARC (adj.PRR 2.76; 95% CI 2.10,3.62; *p* = 0.000), knowledge of site of administration of implants (adj.PRR 1.83; 95% CI 1.17,2.87; *p* = 0.008) and respondents agreeing that their partners should make decisions for their contraceptive to use (adj.PRR 1.49; 95% CI 1.18,1.88; *p* = 0.000). On the other hand, factors associated with reduced use of LARC currently was respondents agreeing that LARC should be used by married women (adj.PRR 0.63; 95% CI 0.44,0.90; *p* = 0.000).

**Table 5:** Multivariable analysis of factors associated with current use of LARC

**OUTCOME: Current use of LARC**				
	**Total**	**Current use of LARC, n (%)**	**Unadjusted (95% CI) PRR**	**Adjusted (95% CI) PRR**
**Age (years)**				
< 24	228	65 (36.31)	1	1
≥ 24	337	114 (63.69)	1.19 (0.92-1.53)	1.01 (0.81-1.27)
**No. of children delivered**				
None	113	26 (14.53)	1	1
Has children	426	153 (85.47)	1.47 (1.03-2.11)	1.20 (0.85-1.68)
**Marital status**				
Never married	113	27 (15.08)	1	1
Ever married	452	152 (84.92)	1.41 (0.99-2.01)	1.12 (0.80-1.58)
**Previous use of LARC**				
No	325	55 (30.73)	1	1
Yes	230	124 (69.27)	3.05 (2.33-4.00)	2.89 (2.19-3.81) ***
**Knowledge of duration of protection from pregnancy**				
**IUD**				
No	178	43 (24.02)	1	1
Yes	387	136 (75.98)	1.46 (1.10-2.02)	1.14 (0.79-1.63)
**Implant**				
No	170	40 (22.35)	1	1
Yes	395	139 (77.65)	1.45 (1.11-2.02)	0.88 (0.60-1.28)
**Knowledge of site of administration**				
**IUD**				
No	136	31 (17.32)	1	1
Yes	429	148 (82.68)	1.51 (1.08-2.12)	0.94 (0.65-1.37)
**Implant**				
No	112	21 (11.73)	1	1
Yes	453	158 (88.27)	1.86 (1.24-2.79)	1.83 (1.17-2.87)
**LARC should be used by married women**				
Disagree	457	156 (87.15)	1	1
Agree	108	23 (12.85)	0.62 (0.43-0.92)	0.63 (0.44-0.90) *
**Partner should decide your contraceptive to use**				
Disagree	293	70 (39.11)	1	1
Agree	272	109 (60.89)	1.68 (1.31-2.16)	1.49 (1.18-1.88) ***
**Health workers should explain contraceptive side effects**				
Disagree	52	11 (6.15)	1	1
Agree	513	168 (93.85)	1.79 (1.01-3.17)	1.45 (0.84-2.49)

Statistically significant at *p < 0.05, ***p < 0.001.

## Discussion

The aim of this study was to assess the level of knowledge and attitudes towards LARC methods as well as factors associated with its use among women of reproductive age attending reproductive health services in Lubaga, division Kampala. Whereas the level of knowledge of specific LARC methods in Lubaga division (Table 2) was slightly higher than that of Kampala district [2], it is still sub-optimal. However across the three LARC methods, women had higher levels of knowledge about injectable contraceptives than implants and IUDs. Knowledge of duration of protection from pregnancy was higher for injectable contraceptives than implant and IUD. Knowledge of site of administration for all LARC methods was also relatively high comparable to the national knowledge levels for the three LARC methods [2] suggesting that the needed FP information is reaching the women in Lubaga. The high level of knowledge of LARC methods in our study may be explained by the highly selective group of females selected from health facilities. Lubaga, a peri-urban setting also has a high number of health units which is likely to increase physical access to FP services including contraceptive counseling. However, there is need for further research into why the high level of knowledge does not translate into actual higher use of LARC in this urban setting [2].

Low use of LARC may be due to misconceptions about causation of permanent infertility (Table 3). A survey in Ghana, revealed that women had high knowledge levels of IUD but cited its side effects as the main reason for non-use [27]. Similarly, studies in Kenya and Nigeria revealed that among injectable and implanon (implant) users respectively, misconceptions [28], side effects [28,29] and husbands’ opposition [28] were main reasons for their discontinuation.

In contrast, a study in Jimma, Ethiopia, revealed that women were least knowledgeable about IUD and more knowledgeable about implants and injectables especially among married couples [30]. The difference in findings could be explained by the fact that the Ethiopian study was conducted among married couples which could have been a highly self-selected group that had jointly agreed to use LARC.

In a study in rural Ethiopia, knowledge of IUD was much lower at 13.1% [31] when compared with knowledge of IUD in this study in Lubaga division. Though knowledge of injectable contraceptives (97.8%) is nearly universal when compared to this study in Lubaga, knowledge of implants (74.4%) was comparable [31]. These study differences in knowledge of IUD and implants could potentially be due to rural–urban differences in socio-demographics and availability of specific LARC methods. Since injectable contraceptives are the predominant LARC method currently used in both studies, this may suggest that injectable contraceptives are the LARC method mostly available. Provision of injectable contraceptives at both community and facility levels when compared to facility provision of implants and IUD in some sub-Saharan countries like Nigeria [32], Madagascar [33], Ethiopia [34] and also Uganda [35,36] could have contributed to high injectable contraceptive knowledge and current use.

Nearly half (48.1%) of the women in this study thought that their male partners should decide on the contraceptive to use (Table 3) suggesting that socio-cultural perceptions play an important part in the contraceptive choices made. A study in Kenya and Ethiopia found that partner approval [37] and husband support [38] influences use of FP services and contraceptives respectively. Our study in Lubaga had similar findings on partner’s decision on spouses’ choice of contraceptive. Partner decision was associated with women’s contraceptive use similar to findings from Ghana, Nigeria and Rwanda where partner approval, partner’s support [39,40] or partner objections [41-43] towards contraceptive choice influenced contraceptive use. In South Africa, women that strongly agreed to male decision making regarding child-bearing did not influence effective contraception [44]. In Uganda [45,46] and Burkina Faso [47], scientific evidence has also shown that positive influence of male partners may affect maternal and child health outcomes. Nearly half of the women in this study thought that their male partners should decide the contraceptive to use therefore socio-cultural perceptions in Lubaga especially related to roles of males and females in decision making in families might explain these attitudes among women.

In Nigeria, [48] and Bangladesh [42], acceptors of long acting contraception were more likely to be married women. A study in Nigeria consistent with our study showed that almost half of the married women had previously used IUD and injectables [49]. Since comparable proportions of use of LARC in all three studies were married women, this may suggest that they may have good attitudes towards using LARC methods.

The findings of our study should be interpreted in light of some limitations; the study was cross-sectional therefore we could not establish temporality between current use of LARC and the independent factors. Since the study relied on the respondents’ self-report, there could have been potential for recall bias about the history related to use of LARC methods. We attempted to control for potential confounders of known factors in the multivariable analysis. Misclassification bias may have been introduced when collapsing attitude items from a 5 to 3 point likert scales, the new categorization assumed that those reporting “unsure” disagreed. However, given the small number of responses in the “unsure” category this is unlikely to have greatly biased the results. Suggestion for future research could include conducting qualitative data collection methods in order to adequately assess behaviors of women associated with contraceptive use.

## Conclusion

This study revealed a relatively low level of current use of LARC among women attending clinics in Lubaga, Kampala. Knowledge of site of administration of implants, previous use, and women’s attitude about the important role of male partners in their choice of contraceptives was associated with current use of LARC. Strategies to strengthen client education may be integrated within reproductive health programs in Lubaga division in order to dispel possible myths about LARC. Involving males in the decision making process of contraceptives may also be integrated into strategies to promote family planning services.

## Competing interests

The authors declare that they have no competing interests.

## Authors’ contributions

RA was the principal investigator, originated the study idea, developed the protocol and manuscript drafting and also applied for the FHRDC grant. RT reviewed the study protocol, contributed significantly to statistical analysis and manuscript drafting. JS mentored the author, supervised the manuscript development and revised the methodology. VZ, TS and CM contributed to statistical analysis of the study. DS was the primary supervisor of the author and contributed to statistical analysis. All authors read and approved the final manuscript.
